# Detecting apathy in patients with cerebral small vessel disease

**DOI:** 10.3389/fnagi.2022.933958

**Published:** 2022-08-03

**Authors:** Xiaoping Cai, Hóngyi Zhào, Zhiyi Li, Yu Ding, Yonghua Huang

**Affiliations:** ^1^Department of Neurology, The Seventh Medical Center of Chinese PLA General Hospital, Beijing, China; ^2^7th Department of Health Cadre, The Second Medical Center of Chinese PLA General Hospital, Beijing, China; ^3^Department of Neurology, NO 984 Hospital of PLA, Beijing, China

**Keywords:** actigraphy, apathy, small vessel disease, sleep, neuropsychiatric disorders

## Abstract

**Background:**

Apathy is attracting more and more attention in clinical practice. As one of the most common features of cerebral small vessel disease (CSVD), the assessment of apathy still mainly relies on observers. With the development of Information and Communication Technologies (ICTs), new objective tools take part in the early detection of apathy.

**Objectives:**

To detect apathy in patients with CSVD and find out the relationship between apathy and actigraphic data sampled from the diurnal and nocturnal periods.

**Methods:**

A total of 56 patients with CSVD were recruited for a cross-sectional observational study. Apathy was diagnosed by the diagnostic criteria for apathy in neurocognitive disorders. The presence of lacunes, white matter hyperintensities, cerebral microbleeds (CMBs), and perivascular spaces (PVS) in magnetic resonance imaging (MRI) images were rated independently. Actigraph devices were worn in the non-dominant hands of each subject for 7 consecutive days to collect samples of raw data, and diurnal vector magnitude (VM) and a series of sleep quality variables were obtained.

**Results:**

We found that the frequency of apathy in Chinese patients with CSVD reached 37.50%. Patients in the Apathy+ group showed more lacunes and CMBs, and higher Fazekas scores in comparison to apathy-group individuals. Diurnal VM, instead of other sleep quality variables, was lower in CSVD patients with apathy relative to those without apathy. Lastly, we discovered that diurnal VM and total time in bed (TTB) correlated negatively with apathy severity in patients with CSVD.

**Conclusion:**

Actigraphy is a promising choice to evaluate apathy in patients with CSVD.

## Introduction

Apathy, defined as a disorder of goal-directed behavior, has attracted more and more attention in aging neuroscience (Hachinski et al., 2022). It has been evidenced that apathy is be found in many neurodegenerative disorders, such as Alzheimer’s disease (AD), Parkinson’s disease (PD), and multiple sclerosis (MS) (Zhao et al., 2012; Niino et al., 2014; Brown et al., 2019), and apathy has been confirmed to be correlated with cognitive impairments in cross-sectional studies (Niino et al., 2014; Yu et al., 2020). Several longitudinal studies demonstrated that an obvious decline in cognitive function and daily living ability was accompanied by apathy (Robert et al., 2006; Lechowski et al., 2009). For example, a 4-year prospective longitudinal study recently discovered that a higher level of apathy at baseline enables the prediction of a progressive cognitive decline at 4-year follow-up in patients with MS (Raimo et al., 2022). In most previous research, apathy was diagnosed and assessed by scales such as the Neuropsychiatric Inventory (NPI), the Apathy Evaluation Scale (AES), the Apathy Inventory (AI), and the Geriatric Depression Scale (GDS) (David et al., 2012; Kuhlmei et al., 2013; Zhao et al., 2014; Tay et al., 2019). In Robert et al. (2018), Miller et al. (2021), the diagnostic criteria for apathy in neurocognitive disorders were discussed actively by experts from academia, industry, and regulatory bodies all around the world. In line with the evolvement of the diagnostic criteria of apathy, unreliable self-reporting has been proposed to be substituted and improved by informant-based observable traits and objective tools.

Cerebral small vessel disease (CSVD) refers to a group of pathological processes that affect the small arteries, arterioles, venules, and capillaries of the brain (Pantoni, 2010). CSVD is quite common in the older population and is featured as cognitive deficits, gait abnormalities, and affective disorders (Zhào et al., 2020). A recent systematic review revealed that CSVD affects all major domains of cognitive ability (Hamilton et al., 2021). Our previous study showed that the prevalence of apathy reached 37.3% in older Chinese patients with CSVD, with a lack of goal-directed behavior and diminished goal-directed activities in the dimension of behavior/cognition according to the diagnostic criteria for apathy revised in 2018 (Zhào et al., 2021).

Until now, various assessment scales for apathy have been developed, whereas, the detection of apathy is still debated. This phenomenon can be explained by the fact that the application of scales is rather limited, owing to its dependency on the human observer. Based on the development of novel technologies, apathy experts recommended objective ways such as Information and Communication Technologies (ICTs) on evaluating apathy in coordination with subjective inventories (Robert et al., 2013). For example, the ECOCAPTURE@HOME protocol was reported to assess apathy remotely in patients with AD and Frontotemporal Dementia (FTD) recently (Batrancourt et al., 2019; Godefroy et al., 2021).

Actigraph devices, progressing from piezoelectric to captive sensors, from epoch-based count data to raw data sampled at high frequency, are a potential candidate for ICTs of that use (König et al., 2014). Actigraphy was confirmed to measure sleep quality and daytime activity in our recently published paper (Zhao et al., 2022). Thus, we sought to detect apathy in patients with CSVD and found out the relationship between apathy and actigraphic data sampled from the diurnal and nocturnal periods.

## Methods

### Participants

We launched a cross-sectional observational study from 1 November 2021 to 20 April 2022 and recruited 56 elderly patients with CSVD consecutively from the Department of Neurology at the Seventh Medical Center of PLA General Hospital (Beijing, China). Our study was approved by the Academic Ethics Committee of the Biological Sciences Division of PLA General Hospital in Beijing, China.

The exclusion criteria were as follows: patients with major stroke or cerebral bleeding episodes or tremor; other causes of leukoencephalopathy (e.g., immune, demyelination, genetic); use of psychotropic medications; multisystem diseases, such as polyarteritis nodosa, nervous system vasculitis associated with connective tissue disorders, and vasculitis secondary to infectious; arthritis; MRI contraindications; and other neurodegenerative disorders, such as AD, PD, and FTD.

### Magnetic resonance imaging measurements

A 3.0T MRI brain (Discovery MR750; GE Healthcare, United States) scan displayed white matter lesions reflecting the degree of CSVD. A brain MRI (slice and interslice thicknesses of 5 and 1.5 mm, respectively) was carried out as follows: longitdinal relaxation time (T1) fluid-attenuated inversion recovery [transverse relaxation time (TR), 1750 ms; time of echo (TE), 23 ms; T1, 780 ms; field of view (FOV), 24 cm] and T2-weighted imaging (TR, 7498 ms; TE, 105 ms; FOV, 24 cm) sequences. The assessors were blinded to imaging findings.

### Total cerebral small vessel disease burden score

The total CSVD burden score was calculated according to our previous procedure (Zhao et al., 2022). One point was allocated to each of the following MRI parameters: moderate to severe white matter hyperintensities (WMH) (Fazekas score: 2–3), presence of lacunes, cerebral Microbleeds (CMBs), and moderate to severe basal ganglia-perivascular spaces (PVS) (semi-quantitative rating > 1), with total scores ranging from 0 to 4 points. To elucidate whether actigraphic data differed according to CSVD burden score, subjects were also divided by total CSVD burden score.

### Diagnosis and assessment of apathy

Each patient was diagnosed according to the diagnostic criteria for apathy in neurocognitive disorders (Miller et al., 2021). It is different from the diagnostic criteria proposed in 2018 (Robert et al). In fact, in criteria B (symptoms and duration), Dimensions B1, B2, and B3 were listed as diminished initiative, interest, and emotional expression/responsiveness, respectively. Dimension B4 (social interaction) was removed. The patient exhibits at least one symptom in at least two of the three dimensions. Details were listed in Supplementary material 1.

### Neuropsychiatric inventor and global cognitive function test

All participants completed both NPI and mini-mental State Evaluation (MMSE). The NPI (12 subscales) is a structured interview with a caregiver who is familiar with the subject. The overall frequency (1–4) and severity (1–3) are then rated. Scores on each NPI subscale range from 0 to 12, with higher scores indicating more severe symptoms (Zhao et al., 2014). MMSE is a widely used 30-point test that reflects the global cognitive function (Zhào et al., 2019).

### Wrist actigraphy

According to the procedure, each participant was instructed to wear an ActiGraph GT3X+ device (ActiGraph, Pensacola, United States) on their non-dominant wrist for 24 h per day (except when bathing or swimming) for 7 days.

At the end of the wear period, data were downloaded using ActiLife software (ActiGraph, Pensacola, FL, United States). All data files were visually screened for sufficient wear time and then processed for analysis.

### Objective actigraphic variables detection

**FIGURE 1:**
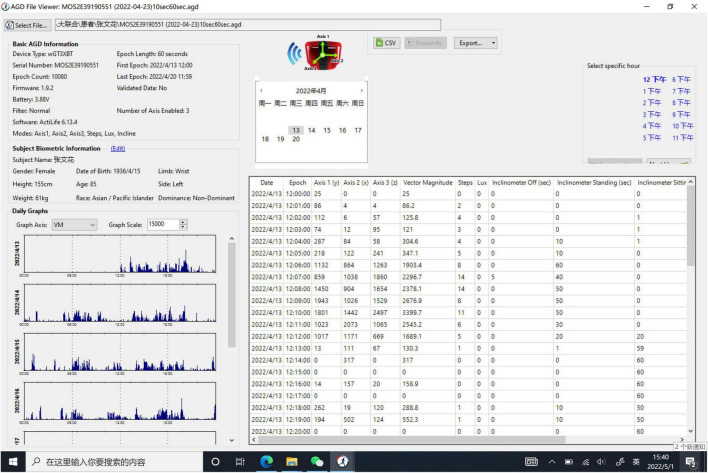
An example of a participant’s actogram, that is, raw actigraphy data of vector magnitude.

Data from the Actigraphic data were downloaded and analyzed using the software with a 60 s epoch. As the ActiGraph GT3X+ device is a triaxial accelerometer, the diurnal vector magnitude (VM) was sampled based on the equation listed as follows: VM = $\sqrt{{\text{X}}^{2}+{\text{Y}}^{2}+{\text{Z}}^{2}}$ (X, Y, and Z are the VM counts at X-axis, Y-axis, and Z-axis, respectively) (Details given in Figure 1). Bedtime and wake time from the sleep diary was used to define sleep-wake variables. The sleep quality variables consisted of sleep efficiency (SE), total time in bed (TTB), total sleep time (TST), wake after sleep onset (WASO), times of awakenings (TA), and average duration of awakenings (ADA) (Zhao et al., 2022).

### Statistical analysis

Differences between the groups’ clinical and demographic data were analyzed by using Student-*t* analysis or one-way analysis of variance. Correlations were determined using the Pearson correlation coefficient to assess the relationship between apathy severity and actigraphic data on the total sample. The significance threshold was set at *p* < 0.05 in all statistical tests. The analysis was carried out using SPSS 22.0 software.

## Results

The frequency of apathy among aged patients with CSVD was 37.50% in this study. Table 1 showed the demographic characteristics of all patients. The gender (66.67% men *vs.* 48.57% men; *p* = 0.268), age (69.24 ± 8.56 years *vs.* 66.11 ± 10.24 years; *p* = 0.246), height (167.14 ± 8.56 cm *vs.* 165.63 ± 6.83cm; *p* = 0.453), weight (72.52 ± 12.47 kg *vs.* 66.31 ± 11.98 kg; *p* = 0.074) and MMSE score (20.95 ± 4.26 *vs.* 23.40 ± 4.72; *p* = 0.057) did not reach significance. Individuals of Apathy+ group and Apathy- group did not show remarked differences in most NPI-subscale scores, except for the NPI-apathy subscale (6.00 ± 1.92 *vs.* 2.06 ± 1.02; *p* < 0.001) and total score (24.80 ± 9.14 *vs.* 16.69 ± 8.57; *p* = 0.002), details were shown in Supplementary material 2. Apathy+ group patients showed more lacunes (mean rank: 23.31 *vs.* 35.06; *p* = 0.004) and CMBs (mean rank: 34.12 *vs.* 24.33; *p* = 0.017), higher Fazekas score (2.09 ± 0.83 *vs.* 1.59 ± 0.74; *p* = 0.023), and higher CSVD burden score (3.24 ± 0.89 *vs.* 2.21 ± 1.27; *p* = 0.0001) compared with Apathy- group individuals. For actigraphic data, diurnal VM were lower in CSVD patients with apathy relative to those without apathy. On the contrary, all the sleep quality variables did not differ statistically between Apathy+ and Apathy- groups. Details are shown in Table 1.

**TABLE 1:** Clinical and demographic characteristics of the subjects with apathy and without apathy.

Characteristics	Apathy + (*N* = 21)	Apathy- (*N* = 35)	Overall (*N* = 56)	P value
Men,%	14(66.67%)	17(48.57%)	31(55.36%)	0.268
Age, years	69.24(8.56)	66.11(10.24)	68.07(10.08)	0.246
Height, cm	167.14(7.95)	165.63(6.83)	166.07(7.48)	0.453
Weight, Kg	72.52(12.47)	66.31(11.98)	68.29(12.42)	0.074
MMSE, score	20.95(4.26)	23.40(4.72)	22.49(4.64)	0.057
SE,%	83.94(8.07)	79.31(7.00)	81.19(7.49)	0.066
TTB, minutes	377.11(109.77)	406.02(110.92)	392.62(114.16)	0.432
TST, minutes	314.54(88.83)	321.90(81.83)	317.63(87.54)	0.793
WASO, minutes	59.47(39.97)	78.40(40.81)	70.42(40.58)	0.164
TA, times	15.16(8.92)	17.12(6.26)	16.18(7.34)	0.425
ADA, minutes	3.73(0.93)	4.84(2.31)	4.42(1.92)	0.054
Diurnal VM, counts	602.39(253.50)	1281.08(348.92)	1026.57(456.67)	0.000***
Lacunes	36.80	22.97	/	0.010**
PVS	28.50	27.71	/	0.450
CMBs	23.94	35.10	/	0.007**
Fazekas score,	2.15(0.81)	1.57(0.74)	1.81(0.80)	0.009**
CSVD burden score	3.35(0.75)	2.17(1.27)	2.65(1.23)	0.001***

Mean (Standard Deviation) for age, height, weight, Fazekas score, and CSVD burden score. Number (Percentage) for gender. Mean rank for LI, PVS, and CMBs. ^**^p < 0.01 Apathy+ relative to Apathy-, ^***^p < 0.001 Apathy+ relative to Apathy-. PVS, enlarged perivascular spaces; CMBs, cerebral microbleeds; CSVD, cerebral small vessel disease; SE, sleep efficiency; TTB, total time in bed; TST, total sleep time; WASO, wake after sleep onset; TA, times of awakenings; ADA, average duration of awakening; VM, vector magnitude.

**FIGURE 2:**
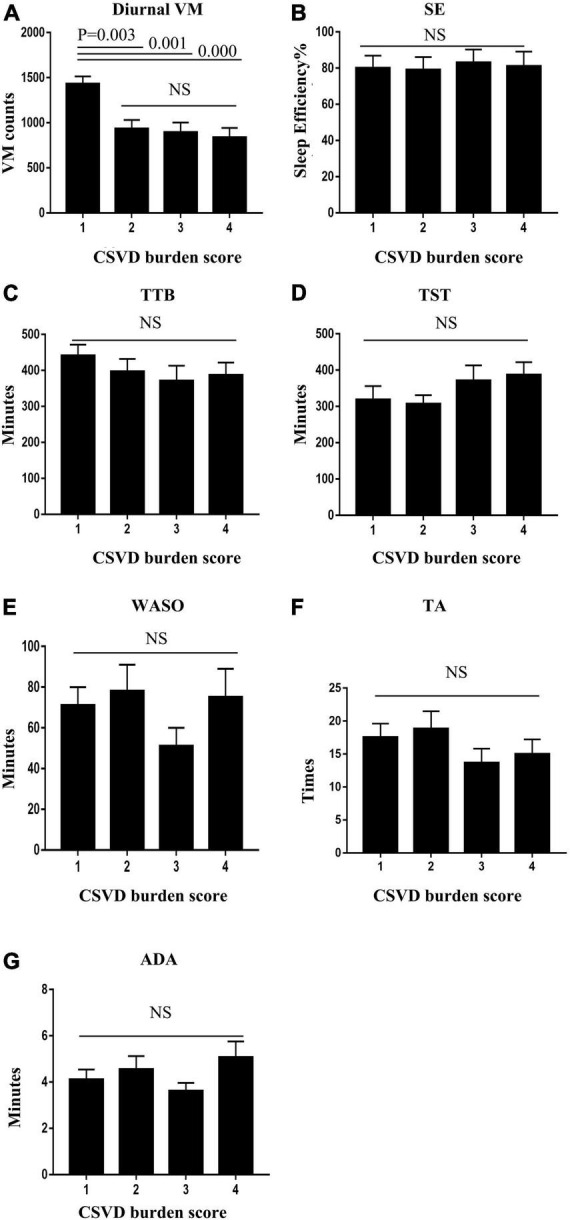
Patients with high cerebral small vessel disease (CSVD) burden (≥ 2 points) exhibited higher diurnal VM relative to those with a low CSVD burden (1 point) **(A)**. Sleep quality variables, including sleep efficiency (SE), total time in bed (TTB), total sleep time (TST), wake after sleep onset (WASO), times of awakenings (TA), and average duration of awakenings (ADA), were not statistically different between groups **(B–G)**.

Patients with a high CSVD burden (≥ 2 points) exhibited lower diurnal VM relative to those with a low CSVD burden (1 point). All the sleep quality variables, including SE, TTB, TST, WASO, TA, and ADA, were not statistically different between groups. Details are shown in Figure 2.

**FIGURE 3:**
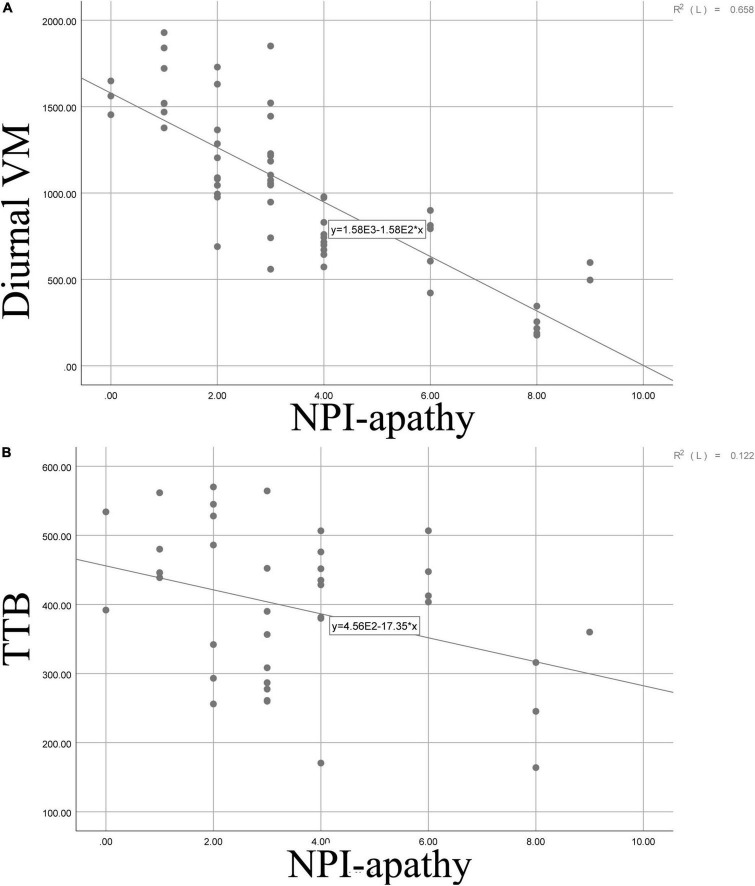
Scatter graphs demonstrating the relationship between apathy and diurnal VM **(A)** as well as between apathy and TTB **(B)**. VM, vector magnitude; SE, sleep efficiency; TTB, total time in bed; TST, total sleep time; WASO, wake after sleep onset; TA, times of awakenings; ADA, average duration of awakenings.

Furthermore, correlation was adopted to analyze the relationship between the NPI–apathy score and actigraphic variables; we found that apathy severity score was negatively associated with diurnal VM score (r = −0.811, *p* = 0.000) and TTB (r = −0.349, *p* = 0.030). Details are shown in Figure 3.

## Discussion

Among patients with CSVD, the current study demonstrated that apathy showed more lacunes, CMBs, and higher Fazekas scores relative to individuals without apathy. Furthermore, diurnal VM rather than sleep quality variables were statistically lower in subjects with apathy in comparison with those without apathy. Meanwhile, patients with a high CSVD burden exhibited lower diurnal VM relative to individuals with a low CSVD burden. Lastly, diurnal VM and total time in bed (TTB) correlated negatively with apathy severity in the total sample.

Although the prevalence of apathy in kinds of neuropsychiatric disorders differs, it is no doubt that apathy is one of the most commonly encountered symptoms in the aged population with high frequency (Yao et al., 2015). In patients with CSVD, together with our previous reports, apathy can be found in more than one-third of hospitalized aged adults (Zhào et al., 2021). Systematic review and meta-analysis confirmed that apathy was associated independently with worse CSVD severity (Clancy et al., 2021), and a hypothesis of “vascular apathy” has been proposed on the ground of the empirical findings that supported the close relationship between apathy and CSVD (Wouts et al., 2020). The hypothesis was based on the inference that apathy symptoms and CSVD share the same functional brain area. In detail, the brain circuits such as the frontal regions with their projections to the prefrontal regions, the basal ganglia, the parietal regions, and the anterior cingulate, which play key roles in planning, motivation, and autoactivation, could be injured in CSVD (Wouts et al., 2020). More recently, apathy, combined with gait impairment and executive dysfunction, was conveyed as a new vascular triad in patients with CSVD by Hachinski et al. (2022). Hypertension, cerebral hypoperfusion, white matter tract disconnection, and other CSVD etiological factors were reported to cause apathy (Moretti et al., 2015; Tay et al., 2019; Hachinski et al., 2022).

Given that individuals with apathy often manifested in those with a lack of goal-directed behavior, there exists several studies that tried to elucidate the definite relationship between apathy and actigraphic data in patients with different neurological disorders. Müller et al. (2006) observed that patients with high apathy exhibited significantly reduced locomotor activity and more episodes of inactivity (naps) during the daytime in patients with traumatic brain injury. David et al. (2012) found that individuals with AD who had symptoms of apathy had significantly lower daytime mean motor activity than AD patients without apathy. In patients with mild cognitive impairment (MCI) and dementia, AES scores correlated negatively with actigraphic daytime activity (Kuhlmei et al., 2013). The present study also concluded that apathy severity was negatively associated with diurnal VM, which implied that lower daytime activity sampled by actigraph devices could be a representation of apathy in patients with CSVD. The difference observed with the other studies using actigraphy was mainly due to the population selection. In addition, the algorithm we used was different from other research, which might be another reason.

Contrary to the findings which revealed that high CSVD burden individuals showed less diurnal VM, all the sleep quality variables did not differ between groups of patients with distinct CSVD burden. This phenomenon is not similar to the findings of Zhào et al. (2021). We considered that these dissimilarities resulted from the sleep evaluation methods we selected. Actigraphy is an objective tool to measure sleep quality, which is different from the subjective questionnaire chosen by Zhao. Pearson correlation demonstrated that apathy was negatively associated with TTB, which is in accordance with the results from patients with AD (Mulin et al., 2011). Taken together, the close relationship between apathy and diurnal activity, as well as TTB, might imply a potential alteration of the sleep/wake circadian rhythm in CSVD patients with apathy. A previous study indicated that abnormalities in sleep/wake circadian rhythm detected by actigraph devices were associated with a high risk of post-stroke apathy (Cosin et al., 2015). It is quite attractive in recent years that patients with CSVD were found to show disrupted 24-h activity rhythm (Zuurbier et al., 2015; Sommer et al., 2021).

Several limitations of this study need to be mentioned. First, apathy was assessed using NPI-apathy, instead of diagnostic criteria of apathy, due to a lack of consensus criteria for apathy diagnosis in CSVD these years. Second, the sample size was not large. In future studies, we need to collect more patients with CSVD.

In conclusion, patients with CSVD have a high incidence of apathy. Actigraphy is a promising choice to evaluate apathy in patients with CSVD.

## Data availability statement

The raw data supporting the conclusions of this article will be made available by the authors, without undue reservation.

## Ethics statement

The studies involving human participants were reviewed and approved by the Academic Ethics Committee of the Biological Sciences Division of PLA General Hospital in Beijing, China. The patients/participants provided their written informed consent to participate in this study.

## Author contributions

XC was responsible for statistical analysis. HZ was responsible for manuscript writing. ZL and YD were responsible for data collection. YH was responsible for studying concepts and design. All authors contributed to the article and approved the submitted version.
