# Supplementary material for: Detecting apathy in patients with cerebral small vessel disease
Source: Front Aging Neurosci. 2022 Aug 3;14:933958. doi: 10.3389/fnagi.2022.933958 (PMC9381828; doi:10.3389/fnagi.2022.933958)
Supplement: Supplementary file 1 [file Data_Sheet_1.docx]

Supplementary Material 1

**For a diagnosis of apathy, the patient needs to meet criteria A, B, C, and D**

**Criterion A.**

Primary diagnoses

The patient meets criteria for a syndrome of cognitive impairment or dementia (as defined by either ICD or DSM-5 criteria; e.g.: AD, vascular dementia, FTD, DLB, PDD, a pre-dementia cognitive impairment syndrome such as MCI, prodromal AD, or other cognitive disorder).

**Criterion B.**

Symptoms and duration

The patient exhibits at least one symptom in at least two of the following three dimensions (B1 to B3). These symptoms have been persistent or frequently recurrent for a minimum of 4 weeks and represent a change from the patient’s usual behavior. These changes may be reported by the patient themselves or by observation of others.

Dimension B1 Diminished initiative: Less spontaneous and/or active than usual self:

- Less likely to initiate usual activities such as hobbies, chores, self-care, conversation, work-related or social activities

Dimension B2 Diminished interest: Less enthusiastic about usual activities:

- Less interested in, or less curious about events in their environment

- Less interested in activities and plans made by others

- Less interested in friends and family

- Reduced participation in activities even when stimulated

- Less persistence in maintaining or completing tasks or activities

Dimension B3 Diminished emotional expression/responsiveness:

- Less spontaneous emotions

- Less affectionate compared to their usual self

- Expresses less emotion in response to positive or negative events

- Less concerned about the impact of their actions on other people

- Less empathy

**Criterion C.**

Exclusionary criteria

These symptoms are not exclusively explained by psychiatric illnesses, intellectual disability, physical disabilities, motor disabilities, change in level of consciousness, or the direct physiological effects of a substance.

**Criterion D.**

Severity

These symptoms cause clinically significant impairment in personal, social, occupational, and/or other important areas of functioning. This impairment must be a change from their usual behaviour.
