# Supplementary material for: Detecting apathy in patients with cerebral small vessel disease
Source: Front Aging Neurosci. 2022 Aug 3;14:933958. doi: 10.3389/fnagi.2022.933958 (PMC9381828; doi:10.3389/fnagi.2022.933958)
Supplement: Supplementary file 2 [file Data_Sheet_2.docx]

**Supplementary Material 2** Clinical and demographic characteristics of the subjects with apathy and without apathy.

| Characteristics | Apathy+  (N=21) | Apathy-  (N=35) | Overall  (N=56) | P value | |
| --- | --- | --- | --- | --- | --- |
| NPI Total Score | 24.80(9.14) | 16.69(8.57) | 19.10(9.59) | | 0.002** |
| Delusion  Hallucinations  Agitation  Depression  Anxiety  Euphoria  Apathy  Disinhibition  Irritability  Aberrant Motor Behavior  Nighttime Behavior Disturbances  Appetite and Eating Abnormalities | 1.65(1.23)  2.00(1.26)  1.80(1.32)  1.85(1.04)  1.65(1.35)  1.65(1.34)  6.00(1.92)  1.30(0.92)  2.05(1.31)  2.15(1.53)  0.75(0.65)  1.95(1.19) | 1.46(1.20)  1.37(1.11)  1.27(1.24)  1.29(1.10)  1.48(1.18)  1.18(1.11)  2.06(1.02)  1.04(0.97)  1.60(1.45)  1.71(1.25)  1.07(0.97)  1.31(1.02) | 1.50(1.17)  1.57(1.17)  1.47(1.27)  1.43(1.11)  1.53(1.21)  1.26(1.25)  3.49(2.37)  1.05(0.99)  1.97(1.46)  1.96(1.46)  0.96(0.83)  1.48(1.13) | | 0.571  0.071  0.234  0.068  0.359  0.131  0.000***  0.247  0.260  0.257  0.197  0.053 |

Mean (Standard Deviation) for all characteristics.

**P＜0.01 Apathy+ relative to Apathy-, ***P＜0.001 Apathy+ relative to Apathy-.
